# LncRNA SNHG5 promotes nasopharyngeal carcinoma progression by regulating miR-1179/HMGB3 axis

**DOI:** 10.1186/s12885-020-6662-5

**Published:** 2020-03-04

**Authors:** Dengtao Liu, Yanpeng Wang, Yigang Zhao, Xiao Gu

**Affiliations:** 1 Clinical laboratory, Linyi People’s Hospital, Linyi, Shandong Province People’s Republic of China; 2 E.N.T. Department, Linyi People’s Hospital, No. 27 East section of Jiefang Road, Linyi, 276000 Shandong Province People’s Republic of China

**Keywords:** SNHG5, Nasopharyngeal carcinoma, miR-1179, HMGB3

## Abstract

**Background:**

Long noncoding RNAs (lncRNAs) have been reported to be important regulators in pathogenesis of human cancers, including nasopharyngeal carcinoma (NPC). Here, we mainly aimed to explore the mechanisms of LncRNA-SNHG5/ miR-1179/HMGB3 axis in NPC progression.

**Methods:**

RT-qPCR and Western blot analysis were employed to detect mRNA and protein expressions. CCK-8, Transwell and dual luciferase reporter assays were applied to investigate functions of LncRNA-SNHG5/miR-1179/HMGB3 axis.

**Results:**

Upregulation of lncRNA-SNHG5 and downregulation of miR-1179 were identified in NPC, which were associated with adverse clinical outcomes. Functionally, upregulation of lncRNA-SNHG5 and downregulation of miR-1179 accelerated NPC cell proliferation, migration and invasion. Furthermore, lncRNA-SNHG5 acted as a molecular sponge of miR-1179 in NPC. Besides that, upregulation of HMGB3 was found in NPC, and knockdown of HMGB3 restrained NPC progression. Moreover, HMGB3, a target of miR-1179, regulated NPC progression by mediating LncRNA-SNHG5/miR-1179 axis.

**Conclusion:**

LncRNA SNHG5 serves as a tumor promoter in NPC by sponging miR-1179 and upregulating HMGB3.

## Introduction

Nasopharyngeal carcinoma (NPC) is one of the most common malignant tumors in China, and the incidence rate is the first of the malignant tumors of the ear, nose and throat [1]. The pathogenesis of NPC is multifaceted and is mainly affected by heredity, viral infection, and environment. Because NPC is sensitive to radiation therapy, radiation therapy is the treatment of choice for NPC patients. However, for advanced cancer and cases of recurrence after radiotherapy, surgical resection and chemical treatment are also indispensable means [2]. Moreover, due to the easy recurrence and early metastasis of tumors, the prognosis of patients with NPC is poor. The 5-year survival rate for patients who are not sensitive to radiation is about 10%, and the radiation-sensitive 5-year survival rate is about 30% [3]. Therefore, it is necessary to explore effective targets to improve the survival rate of NPC patients.

Long noncoding RNAs (lncRNAs) have been demonstrated to serve as tumor promoter or inhibitor in the pathogenesis of human cancers. Furthermore, lncRNA SOX2-OT promoted proliferation and metastasis of NPC cells through miR-146b-5p/HNRNPA2B1 pathway [4]. On the contrary, lncRNA-LET was downregulated in NPC tissues and restrained proliferation and invasion of NPC cells [5]. Recently, the specific roles of lncRNA Small Nucleolar RNA Host Gene 5 (SNHG5) caught our attention. In particularly, upregulation of SNHG5 was identified in melanoma and glioma and function as an oncogene [6, 7]. But SNHG5 was found to be downregulated in gastric cancer and blocked gastric cancer progression by trapping MTA2 [8]. These findings imply that SNHG5 has tissue specificity. Moreover, the dysregulation of SNHG5 remains unknown in NPC. Thus, this study was designed to explain the regulatory mechanism of SNHG5 in NPC.

Increasing studies suggest that lncRNAs involve in tumorigenesis by acting as miRNA sponges [9]. Here, miR-1179 was predicted to have binding sites with SNHG5. Previous studies have shown that the expression level of miR-1179 depends on the type of cancer. For example, miR-1179 was upregulated in esophageal squamous cell carcinoma and promoted cell invasion [10]. In contrast, miR-1179 was downregulated in pancreatic cancer and acted as a tumor suppressor [11]. For NPC, the role of miR-1179 is still unclear, which need to be elucidated. In addition, high mobility group box 3 (HMGB3) was predicted to be a target of miR-1179 in this study. Moreover, the dysregulation of HMGB3 has been found in many human cancers. For example, upregulation of HMGB3 was identified in gastric cancer, and knockdown of HMGB3 inhibited proliferation and migration of gastric cancer cells [12]. Moreover, overexpression of HMGB3 promoted cell proliferation, migration and was associated with poor prognosis in urinary bladder cancer patients [13]. Additionally, it has been reported that inhibition of HMGB3 induced by miR-205-5p inhibited cancer cell aggressiveness and was involved in prostate cancer pathogenesis [14]. However, the relationship between miR-1179 and HMGB3 is unclear in NPC.

The purpose of this study was to preliminarily elucidate the molecular mechanism of SNHG5/miR-1179/HMGB3 pathway in NPC progression. Furthermore, the functions of SNHG5, miR-1179 and HMGB3 were also investigated in NPC. Our results will provide potential targets for NPC treatments.

## Materials and methods

### Clinical tissues

Sixty-four NPC tissues and adjacent normal samples were obtained from patients in Linyi People’s Hospital, who did not receive any treatments prior to surgery. Informed consents were obtained from all NPC patients. This study was approved by the Institutional Ethics Committee of Linyi People’s Hospital.

### Cell culture

Normal nasopharyngeal epithelial cells NP69 (BNCC338439) and NPC cell line C666–1 (BNCC337872) were purchased from BeNa Culture Collection (BNCC, Beijing, China) in February, 2018. NP69 and C666–1 cells are not misidentification and contamination of human cell lines (ExPASy: SIB Bioinformatics Resource Portal, https://www.expasy.org/). These cells were seeded in culture solution (90%RPMI-1640 + 10%FBS) at 37 °C in a humid atmosphere with 5% CO_2_.

### Cell transfection

SNHG5 complementary DNA was synthesized and cloned into the expression vector pcDNA3.1. Effective siRNA oligonucleotides targeting SNHG5 and HMGB3 or miR-1179 mimic, miR-1179 inhibitor was obtained from GenePharma (Shanghai, China). Next, they were transfected into C666–1 cells using Lipofectamine 2000 (Invitrogen/Thermo Fisher Scientifc), respectively. Untreated cells were set as the control (Blank).

### Real-time quantitative PCR (RT-qPCR)

Total RNA was extracted using TRIzol reagent (Invitrogen, USA). RNA was reversely transcript into complementary DNA (cDNA) using PrimeScript RT reagent kit (Takara, Dalian, China). RT-qPCR assay was performing using Real-time PCR Mixture assays (Takara) on ABI 7300 Real-time PCR system (Applied Biosystems, Waltham, MA). SNHG5 and miR-1179 expression were normalized to U6, while HMGB3 was normalized to GAPDH. Their expressions were quantified with the 2^−△△cq^ method.

### Western blot analysis

RIPA lysis buffer (Beyotime) was used to obtain protein samples. Next, 10% SDS-PAGE was used to separate protein. Protein samples were transferred to PVDF membranes. Blocked with 5% non-fat milk, protein samples were incubated overnight at 4 °C with E-cadherin, N-cadherin, Bcl-2, Bax and GAPDH primary antibodies (Abcam, Shanghai, China). Afterwards, secondary antibodies (Abcam, USA) were added to incubate protein samples for 1 h. ECL kit (Beyotime) was used to assess protein bands.

### Cell counting Kit-8 (CCK-8) assay

Transfected C666–1 cells (2 × 10^3^ cells/well) were seeds in 96-well plates. Next, these cells were incubated for 24, 48, 72 or 96 h in RPMI-1640 medium, respectively. Then, 10 μL CCK-8 reagents were used to incubate the cells for 4 h. The medium was discarded and dimethyl sulfoxide was added. After 10 min shaking, OD490 was detected by a microplate reader (Olympus Corp., Tokyo, Japan).

### Transwell assay

Cell invasion was detected in the upper chamber with Matrigel. After 30 min, Transwell upper chamber was added with C666–1 cell suspension (2 × 10^3^ cells/well). Next, RPMI-1640 medium (10% FBS) was added to 24-well plates in lower chamber. After 24 h, 0.1% crystal violet was applied to stain the moved cells. Cell migration experiment was performed without Matrigel. Observation and photographing were performed by a light microscope.

### Flow cytometric analysis

Flow cytometric analysis was adopted to detect apoptosis in NPC. First, transfected C666–1 cells (3 × 10^3^ cell/well) were seed in 6-well plates. After 48 h, trypsin (without EDTA) digestion was used to collect C666–1 cells. We then suspended the collected C666–1 cells in PBS at 4 °C. After discarding PBS, Annexin V-FITC from Annexin V-FITC Apoptosis Detection Kit (Biovision, K101) was used to stain C666–1 cells for 15 mins following with Binding Buffer (1×). After that, C666–1 cells were stained with PI (Sigma-Aldrich, Shanghai, China). Finally, we observed the apoptosis of C666–1 cells using a flow cytometer (BD Biosciences).

### Immunocytochemical assay

The section of NPC tissues were dewaxed, hydrated, and washed twice with PBS for 5 min. After blocking with 5% goat serum (diluted in PBS), we incubated the cells with anti-HMGB3 antibody at 37 °C for 1–2 h. Then, we washed them for three times with PBS for 5 min. Subsequently, we incubated them with the HRP-conjugated goat anti-rabbit secondary antibody at 37 °C for 1 h. After washing 3 times with PBS, DAB mixture was used for color development of this section. The section was washed, counterstained, dehydrated, transparentized and mounted. Images were captured using microscope.

### Dual luciferase reporter assay

The 3′-UTR of wild-type and mutant SNHG5 (wt-SNHG5 and mut-SNHG5) or HMGB3 (wt-HMGB3 and mut-HMGB3) were amplified and then inserted into pmiR-GLO vector (Promega Beijing Biotech Co., Beijing, China). Then, the above reporter plasmids were transfected into C666–1 cells with miR-1179 mimics. After 48 h, activities of firefly luciferase were determined by dual-luciferase reporter assay system (Promega, USA).

### Statistical analysis

Data were analyzed using Student’s t-test or one-way ANOVA in SPSS 19.0 or Graphpad Prism 6. Data are shown as mean ± SD. Univariate Kaplan-Meier method with log-rank test was used to analyze the association between SNHG5 and survival rate. *P* < 0.05 was defined as statistically difference.

## Results

### The dysregulation of LncRNA-SNHG5 and miR-1179 was found in NPC

**Fig. 1:**
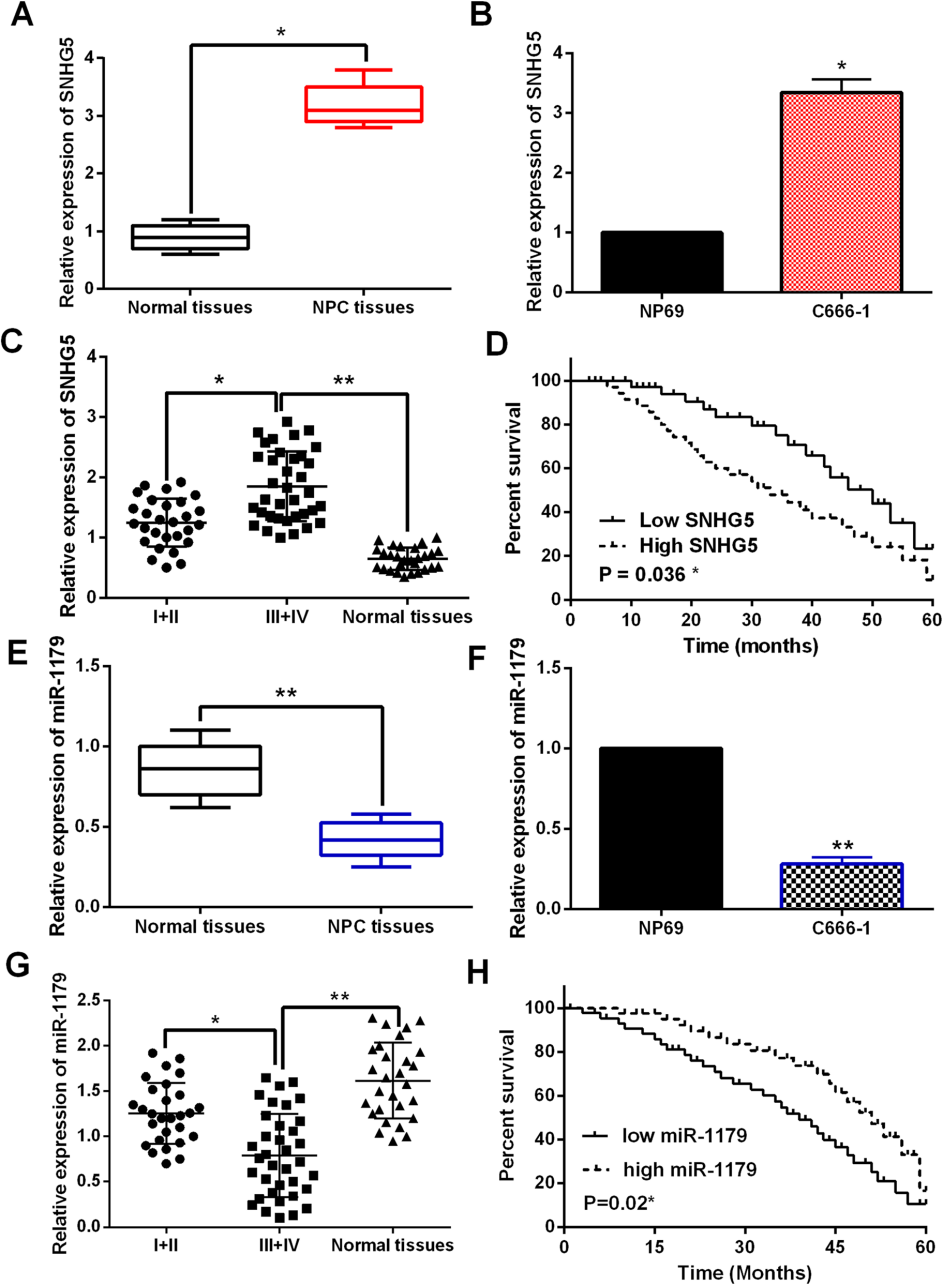
The dysregulation of LncRNA-SNHG5 and miR-1179 was found in NPC. **a** The LncRNA-SNHG5 expression in NPC tissues and normal tissues **b** LncRNA-SNHG5 expression in C666–1 and NP69 cells **c** LncRNA-SNHG5 expression in different clinical stage of NPC patients **d** High LncRNA-SNHG5 expression patients predicted worse prognosis. **e** The miR-1179 expression in NPC tissues and normal tissues **f** MiR-1179 expression in C666–1 and NP69 cells **g** MiR-1179 expression in different clinical stage of NPC patients **h** Low miR-1179 expression patients predicted worse prognosis. **P < 0.05, **P < 0.01

The abnormal expressions of LncRNA-SNHG5 and miR-1179 were detected in NPC tissues. First, LncRNA-SNHG5 expression was increased in NPC tissues compared to normal tissues (Fig. 1a). Similarly, upregulation of LncRNA-SNHG5 was also identified in C666–1 cells compared with NP69 cells (Fig. 1b). Additionally, higher LncRNA-SNHG5 expression was found in advanced stages than in early stages of NPC patients (Fig. 1c). And poor prognosis in NPC patients was related to high LncRNA-SNHG5 expression (Fig. 1d). Next, we found that miR-1179 expression was downregulated in NPC tissues contrast to normal tissues (Fig. 1e). Furthermore, miR-1179 was also decreased in C666–1 cells compared with NP69 cells (Fig. 1f). Moreover, miR-1179 was much lower in advanced stages of NPC patients than in early stages (Fig. 1g). Furthermore, downregulation of miR-1179 was associated with shorter overall survival in NPC patients (Fig. 1h). Based on these results, we suspect that LncRNA-SNHG5 and miR-1179 may be affected the tumorigenesis of NPC.

### LncRNA-SNHG5 acted as a molecular sponge of miR-1179 in NPC

**Fig. 2:**
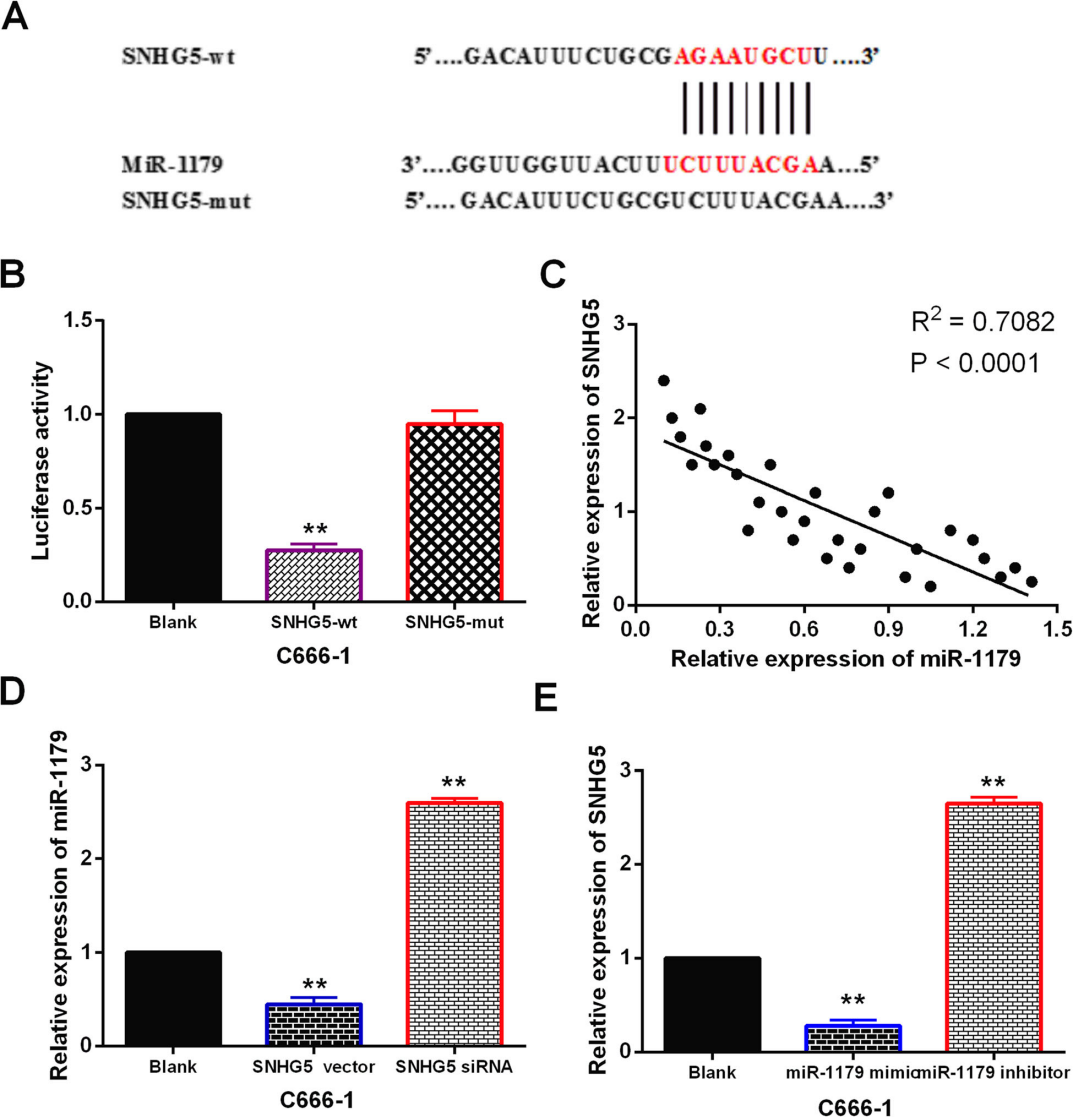
LncRNA-SNHG5 acted as a molecular sponge of miR-1179 in NPC. **a** The binding sites between LncRNA-SNHG5 and miR-1179. **b** Luciferase reporter assay **c** MiR-1179 was negatively correlated with LncRNA-SNHG5 expression in NPC tissues. **d** MiR-1179 expression regulated by si-SNHG5 and SNHG5 vector in C666–1 cells **e** SNHG5 expression in C666–1 cells containing miR-1179 mimics or inhibitor ** *P* < 0.01

Next, miR-1179 was predicted to have binding sites with LncRNA-SNHG5 in starBase version 2.0 (http://starbase.sysu.edu.cn/, Fig. 2a). Dual luciferase reporter was then performed to verify the above prediction. We found that miR-1179 mimics obviously reduced the luciferase activity of wt-SNHG5, but had little effect on mut-SNHG5 in C666–1 cells (Fig. 2b). Moreover, LncRNA-SNHG5 was found to inversely regulate miR-1179 expression in NPC tissues (Fig. 2c). Next, miR-1179 expression was observed in C666–1 cells with si-SNHG5 and SNHG5 vector. RT-qPCR showed that upregulation of SNHG5 decreased miR-1179 expression, while knockdown of SNHG5 increased miR-1179 expression (Fig. 2d). At the same time, how miR-1179 regulates LncRNA-SNHG5 expression was also detected in C666–1 cells. LncRNA-SNHG5 expression was also reduced by miR-1179 mimics and enhanced by miR-1179 inhibitor (Fig. 2e). These results imply that LncRNA-SNHG5 acts as a molecular sponge of miR-1179 in NPC.

### LncRNA-SNHG5/miR-1179 axis was involved in NPC progression

**Fig. 3:**
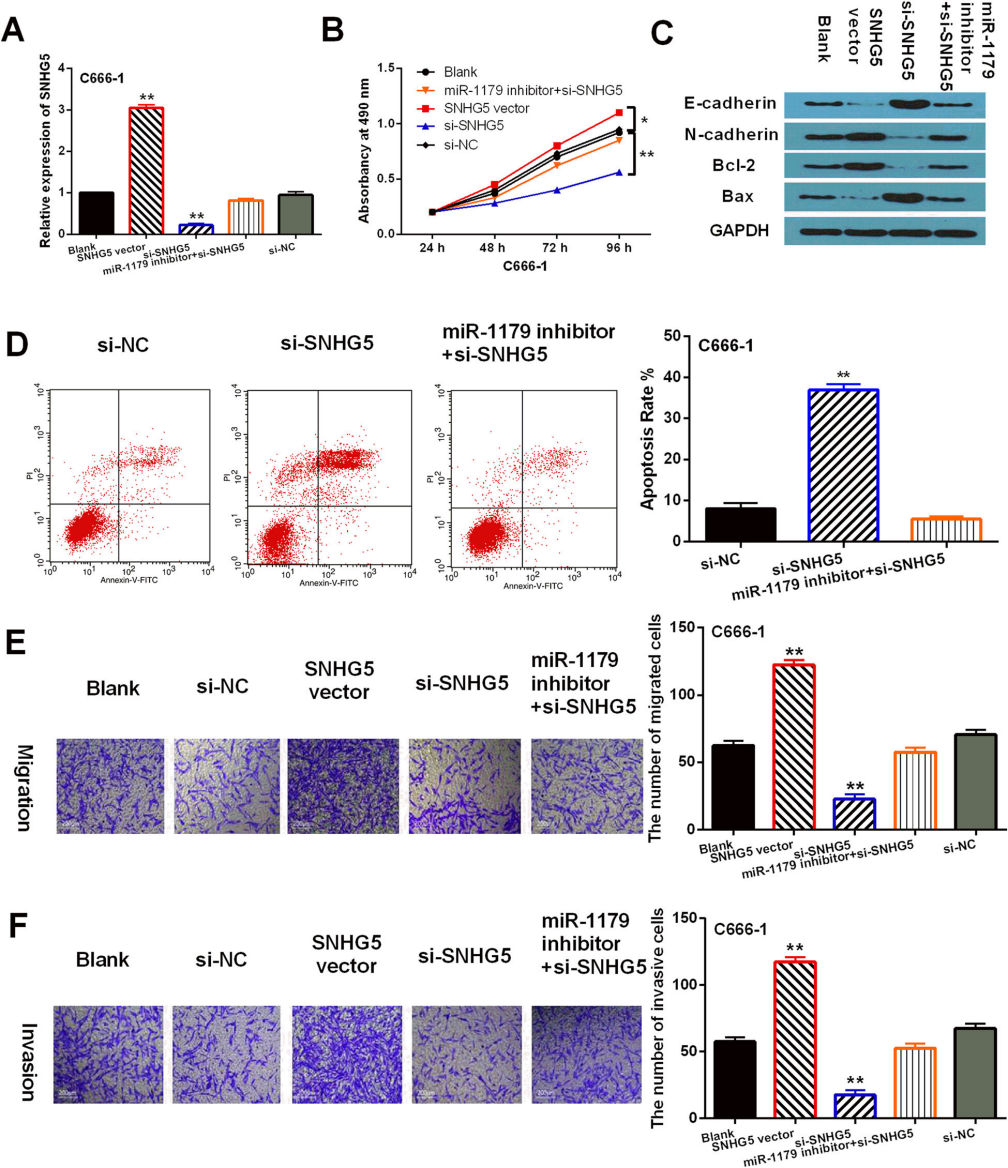
LncRNA-SNHG5 upregulation promoted NPC progression. **a** LncRNA-SNHG5 expression in C666–1 cells with si-SNHG5, SNHG5 vector or si-SNHG5 + miR-1179 inhibitor. **b**, **e**, **f** Cell proliferation, migration, invasion in C666–1 cells with si-SNHG5, SNHG5 vector or si-SNHG5 + miR-1179 inhibitor. **c** The protein expressions of E-cadherin, N-cadherin, Bax and Bcl-2 were detected by Western Blot analysis in C666–1 cells with si-SNHG5, SNHG5 vector or si-SNHG5 + miR-1179 inhibitor. Full-length blots/gels are presented in Supplementary Figure 3C. **d** Apoptosis in C666–1 cells with si-SNHG5 or si-SNHG5 + miR-1179 inhibitor **P < 0.05, ** *P* < 0.01

To further assess the regulatory mechanism of LncRNA-SNHG5/miR-1179 in NPC, miR-1179 mimics, miR-1179 inhibitor, si-SNHG5 or SNHG5 vector was transfected into C666–1 cells, respectively. RT-qPCR indicated that LncRNA-SNHG5 expression was downregulated by si-SNHG5 and upregulated by SNHG5 vector in C666–1 cells. After transfection of miR-1179 inhibitor, the decreased expression of LncRNA-SNHG5 induced by si-SNHG5 was recovered (Fig. 3a). Functionally, CCK-8 assay suggested that upregulation of LncRNA-SNHG5 promoted cell proliferation, while knockdown of LncRNA-SNHG5 restrained proliferation of C666–1 cells. And miR-1179 inhibitor abolished the inhibitory effect of si-SNHG5 on C666–1 cell proliferation (Fig. 3b). Additionally, downregulation of LncRNA-SNHG5 promoted E-cadherin and Bax expression and inhibited N-cadherin and Bcl-2 expressions, while LncRNA-SNHG5 overexpression showed opposite effect on these genes in C666–1 cells. Similarly, miR-1179 inhibitor also exerted reverse effect on these genes regulated by si-SNHG5 (Fig. 3c). We also found that knockdown of LncRNA-SNHG5 induced apoptosis of C666–1 cells. Furthermore, miR-1179 inhibitor abolished the effect of LncRNA-SNHG5 on apoptosis in C666–1 cells (Fig. 3d). In addition, Transwell assay showed that cell migration and invasion were promoted by SNHG5 vector and restrained by si-SNHG5. Furthermore, si-SNHG5 mediated inhibition of cell migration and invasion was also restored by miR-1179 inhibitor (Fig. 3e, f). These results reveal that LncRNA-SNHG5 serves as a tumor promoter in NPC by targeting miR-1179.

**Fig. 4:**
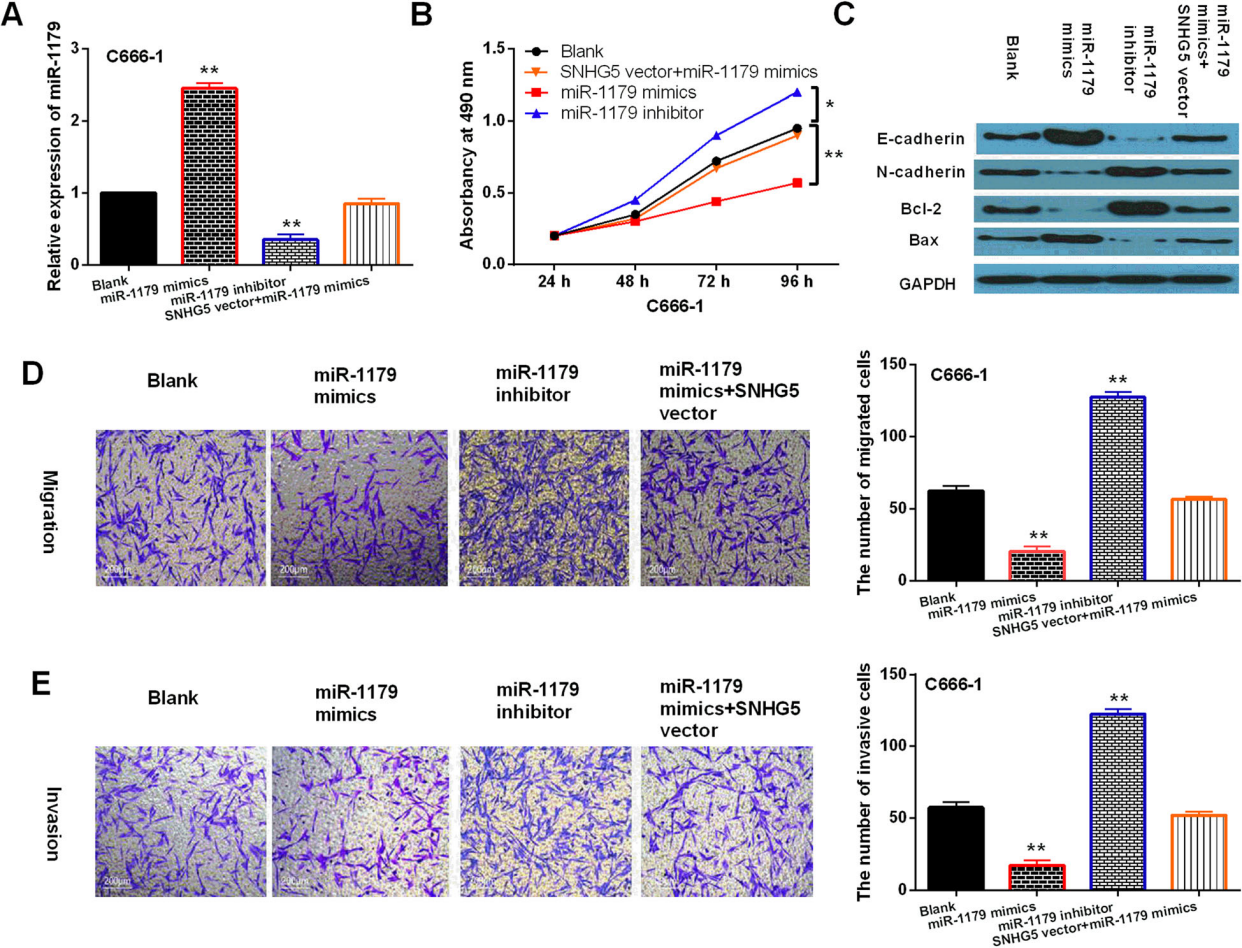
Overexpression of miR-1179 inhibited NPC progression. **a** MiR-1179 expression in C666–1 cells with miR-1179 mimics or inhibitor and SNHG5 vector+miR-1179 mimics. **b**, **d**, **e** Cell proliferation, migration and invasion in C666–1 cells with miR-1179 mimics or inhibitor and SNHG5 vector+miR-1179 mimics. **c** The protein expressions of E-cadherin, N-cadherin, Bax and Bcl-2 were detected by Western Blot analysis in C666–1 cells with miR-1179 mimics or inhibitor and SNHG5 vector+miR-1179 mimics. Full-length blots/gels are presented in Supplementary Figure 4C. **P < 0.05, ** *P* < 0.01

Besides that, we found that miR-1179 mimics declined its expression, whereas miR-1179 inhibitor enhanced its expression in C666–1 cells. However, SNHG5 vector reduced the increased expression of miR-1179 induced by its mimics (Fig. 4a). Functionally, cell proliferation, migration and invasion were restrained by miR-1179 overexpression and accelerated by downregulation of miR-1179. And the reverse effect of SNHG5 vector on miR-1179 mimics mediated inhibition of cell proliferation, migration and invasion was also found in C666–1 cells (Fig. 4b, d, e). Additionally, miR-1179 mimics was found to promote E-cadherin and Bax expression and restrain N-cadherin and Bcl-2 expressions, while miR-1179 inhibitor exerted opposite effect. Similarly, SNHG5 vector also exerted reverse effect on these genes regulated by miR-1179 mimics in C666–1 cells (Fig. 4c). The results suggest that miR-1179 functions as a tumor inhibitor in NPC by interacting with LncRNA-SNHG5.

### HMGB3 is a direct target of miR-1179

**Fig. 5:**
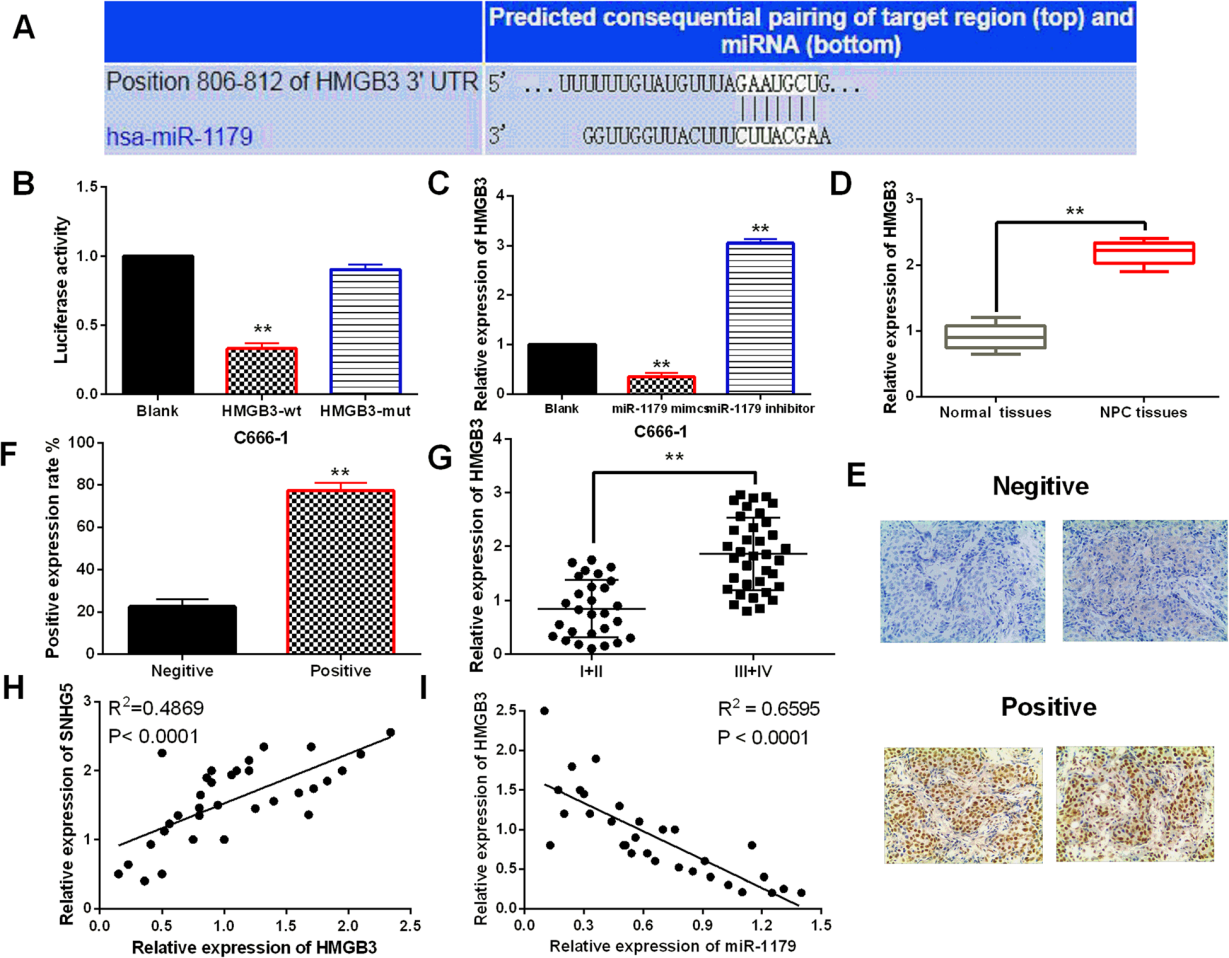
HMGB3 is a direct target of miR-1179. **a** The binding sites between HMGB3 and miR-1179. **b** Luciferase reporter assay **c** HMGB3 expression regulated by miR-1179 mimics or inhibitor in C666–1 cells **d** HMGB3 expression in NPC tissues and normal tissues. **e**, **f** The protein expression of HMGB3 in NPC tissues detected by immunohistochemistry. **g** HMGB3 expression in different clinical stage of NPC patients. **h** SNHG5 was positively correlated with HMGB3 expression in NPC tissues. **i** HMGB3 was negatively correlated with miR-1179 expression in NPC tissues. ** *P* < 0.01

MiRNAs are well-known to involve in tumorigenesis by binding to target genes. TargetScan (http://www.targetscan.org) predicted that miR-1179 has binding sites with the 3′-UTR of HMGB3 (Fig. 5a). Luciferase reporter assay indicated that miR-1179 mimics declined wt-HMGB3 luciferase activity, but had no effect on mut-HMGB3 (Fig. 5b). At the same time, we found that HMGB3 expression was downregulated by miR-1179 mimics and upregulated by miR-1179 inhibitor (Fig. 5c). Moreover, HMGB3 was found to be upregulated in NPC tissues compared to normal tissues (Fig. 5d). The results of IHC displayed positive detection of HMGB3 protein expressions in the nucleus of NPC tissue (Fig. 5e). Moreover, the protein expression intensity of HMGB3 was obviously increased in NPC tissues compared to the adjacent normal tissues (Fig. 5f). And higher HMGB3 expression was identified in advanced stages of NPC patients than in early stages (Fig. 5g). Additionally, a negative correlation between HMGB3 and miR-1179 expressions was identified in NPC tissues (Fig 5h). But LncRNA-SNHG5 was found to positively regulate HMGB3 expression in NPC tissues (Fig. 5i). These results indicated that HMGB3 is a direct target of miR-1179.

### HMGB3 regulated NPC progression by mediating LncRNA-SNHG5/miR-1179 axis

**Fig. 6:**
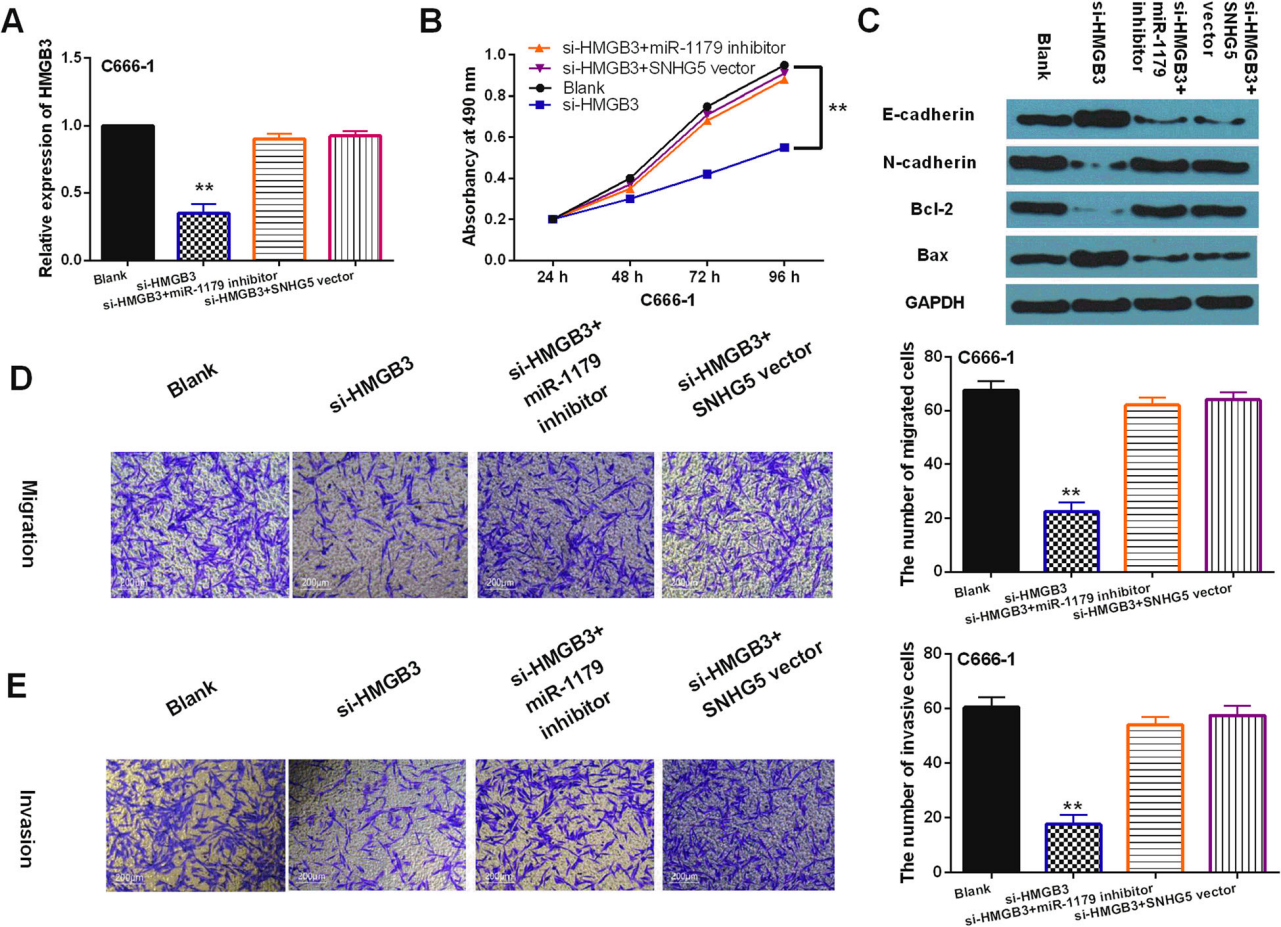
HMGB3 regulated NPC progression by mediating LncRNA-SNHG5/miR-1179 axis. **a** HMGB3 expression in C666–1 cells with si-HMGB3, si-HMGB3 + miR-1179 inhibitor or si-HMGB3 + SNHG5 vector. **b**, **d**, **e** Cell proliferation, migration and invasion in C666–1 cells with si-HMGB3, si-HMGB3 + miR-1179 inhibitor or si-HMGB3 + SNHG5 vector. **c** The protein expressions of E-cadherin, N-cadherin, Bax and Bcl-2 were detected by Western Blot analysis in C666–1 cells with si-HMGB3, si-HMGB3 + miR-1179 inhibitor or si-HMGB3 + SNHG5 vector. Full-length blots/gels are presented in Supplementary Figure 6C. *P < 0.05, ** *P* < 0.01

To further explore the interaction between HMGB3 and LncRNA-SNHG5/miR-1179 axis, SNHG5 vector or miR-1179 inhibitor was transfected into C666–1 cells with si-HMGB3. First, we found that si-HMGB3 significantly reduced its expression in C666–1 cells. But SNHG5 vector or miR-1179 inhibitor recovered this reduction of HMGB3 (Fig. 6a). Functionally, knockdown of HMGB3 restrained proliferation of C666–1 cells, while SNHG5 vector or miR-1179 inhibitor recovered si-HMGB3 mediated inhibition of cell proliferation (Fig. 6b). Moreover, knockdown of HMGB3 was found to promote E-cadherin and Bax expression and restrain N-cadherin and Bcl-2 expressions in C666–1 cells. SNHG5 vector or miR-1179 inhibitor also reversely regulated the effect of si-HMGB3 on these genes (Fig. 6c). In addition, cell migration and invasion were found to be restrained by downregulation of HMGB3. And SNHG5 vector or miR-1179 inhibitor abolished the suppressive effect of si-HMGB3 on C666–1 cell migration and invasion (Fig. 6d, e). Collectively, LncRNA-SNHG5/miR-1179 axis promoted NPC progression by regulating HMGB3 expression.

## Discussion

Recently, many lncRNAs have been reported to involve in tumorigenesis of NPC. For example, LncRNA-LINC00460 facilitated NPC tumorigenesis through sponging miR-149-5p to up-regulate IL6 [15]. In this study, LncRNA-SNHG5 was found to promote NPC progression by mediating miR-1179/HMGB3 axis. Specifically, upregulation of lncRNA-SNHG5 was identified in NPC, which was associated with clinical stages and poor prognosis. Functionally, upregulation of lncRNA-SNHG5 was found to accelerate proliferation, migration and invasion of NPC cells. Consistent with our results, it was reported that lncRNA-SNHG5 was up-regulated and served as a prognostic biomarker in acute myeloid leukemia [16]. Moreover, lncRNA-SNHG5 has been demonstrated to facilitate osteosarcoma cell proliferation, migration and invasion [17], which was the same as our results. These findings imply that lncRNA-SNHG5 was upregulated in NPC and serves as a tumor promoter in NPC progression.

Many studies have demonstrated that lncRNAs function as miRNA sponges to participate in tumorigenesis. LncRNA-SNHG5 has been found to promote the development of colorectal cancer by sponging miR-132-3p/CREB5 [18]. In our research, lncRNA-SNHG5 was confirmed as a molecular sponge of miR-1179. In addition, miR-1179 was downregulated in NPC and associated with adverse clinical outcomes in NPC patients. Furthermore, overexpression of miR-1179 inhibited NPC cell proliferation, migration and invasion. Similarly to our results, miR-1179 expression was decreased and restrained glioblastoma cell proliferation and cell cycle progression [19]. And miR-1179 was also found to suppress cell growth and invasion by targeting SPAG5 in human non-small cell lung cancer [20]. Besides that, the interaction between lncRNA-SNHG5 and miR-1179 was identified in NPC progression. LncRNA-SNHG5 was found to promote NPC development by sponging miR-1179, which has not been reported in previous studies. All these results demonstrate that miR-1179 acts as a tumor inhibitor in NPC.

Next, we found that HMGB3 is a direct target of miR-1179. The dysregulation of HMGB3 has been identified in many human cancers. For example, increased expression of HMGB3 was found in esophageal squamous cell carcinoma, which predicted worse outcome [21]. Additionally, HMGB3 was found to be upregulated in glioblastoma and promoted cell proliferation and metastasis [22]. Here, upregulation of HMGB3 was identified in NPC and associated with advanced tumor stage in NPC patients. Furthermore, knockdown of HMGB3 suppressed NPC development. Our results are consistent with the above findings. Based on these findings, HMGB3 was confirmed to act as an oncogene in NPC. Besides that, we found miR-1179 inhibited NPC progression by downregulating HMGB3. Similarly, miR-758 was reported to inhibit proliferation, migration, invasion of non-small cell lung cancer cells by negatively regulating HMGB3 [23]. More importantly, lncRNA-SNHG5 was found to facilitate NPC development by upregulating HMGB3, which has not been investigated in NPC. Combining these results, we consider that lncRNA-SNHG5 functions as a tumor promoter in NPC by regulating miR-1179/HMGB3 axis.

## Conclusion

In conclusion, LncRNA-SNHG5 promoted NPC progression by sponging miR-1179 and upregulating HMGB3. It indicates that LncRNA-SNHG5/ miR-1179/HMGB3 pathway may possess a potential power in NPC treatments. Nevertheless, we only initially explained the regulatory mechanism of LncRNA-SNHG5 in NPC. The regulation network of lncRNAs is complex, and we still need to further explore the functional mechanism of SNHG5 in NPC.

## Supplementary information

**Additional file 1.**

**Additional file 2.**

**Additional file 3.**

## Data Availability

The datasets used and/or analyzed during the present study are available from the corresponding author on reasonable request.

## Abbreviations

CCK-8: Cell counting kit-8
cDNA: Complementary DNA
HMGB3: High mobility group box 3
lncRNA: long non-coding RNA
miRNA: microRNA
mRNA: messenger-RNA
Mut: Mutant type
NPC: Nasopharyngeal carcinoma
RT-qPCR: Real-time quantitative PCR
SNHG5: Small Nucleolar RNA Host Gene 5
Wt: Wide type
