# Supplementary figures and images for: LncRNA SNHG5 promotes nasopharyngeal carcinoma progression by regulating miR-1179/HMGB3 axis
Source: BMC Cancer. 2020 Mar 4;20:178. doi: 10.1186/s12885-020-6662-5 (PMC7057527; doi:10.1186/s12885-020-6662-5)

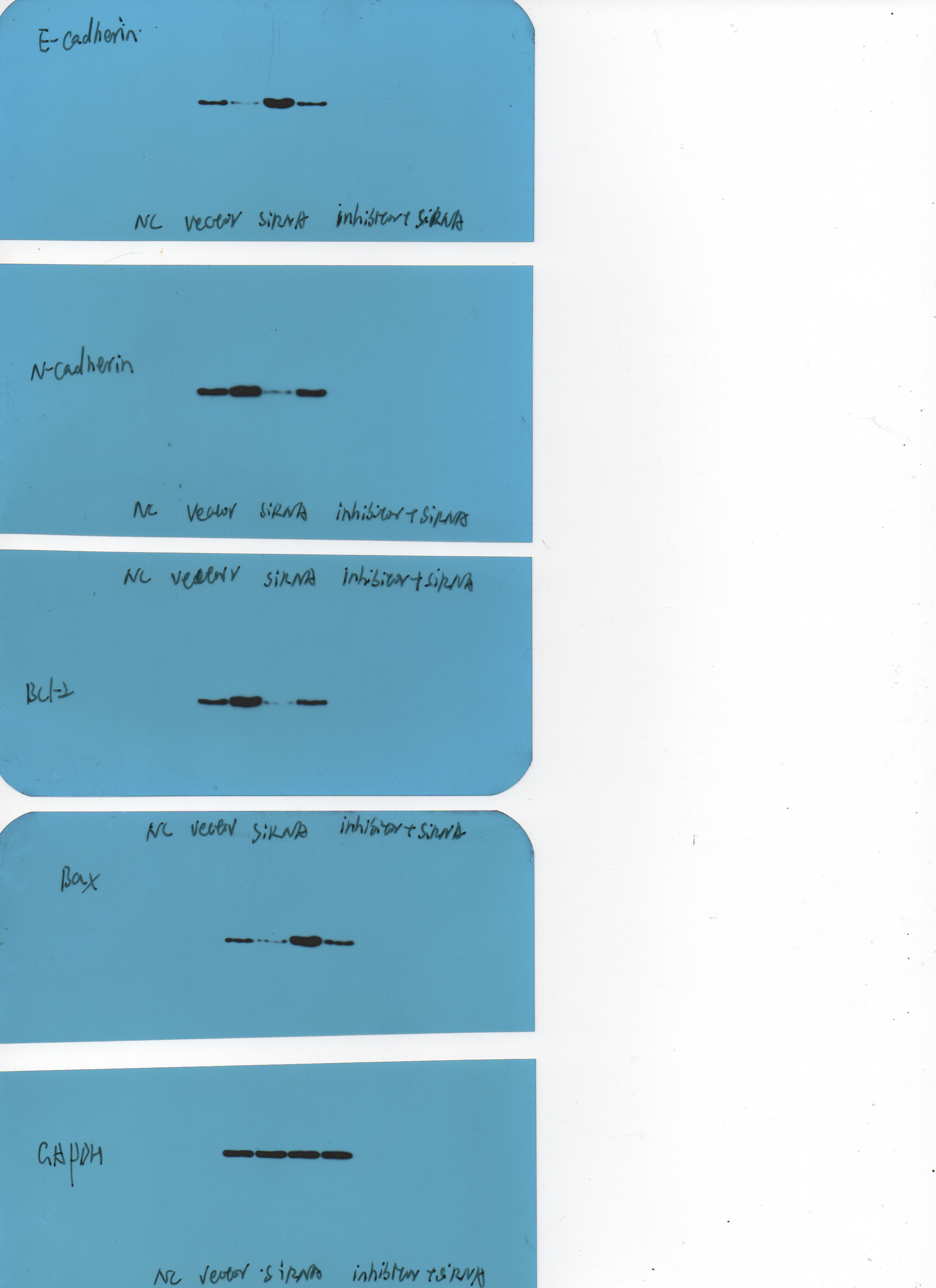

Supplement: Supplementary file 1 — Additional file 1. [file 12885_2020_6662_MOESM1_ESM.tif]

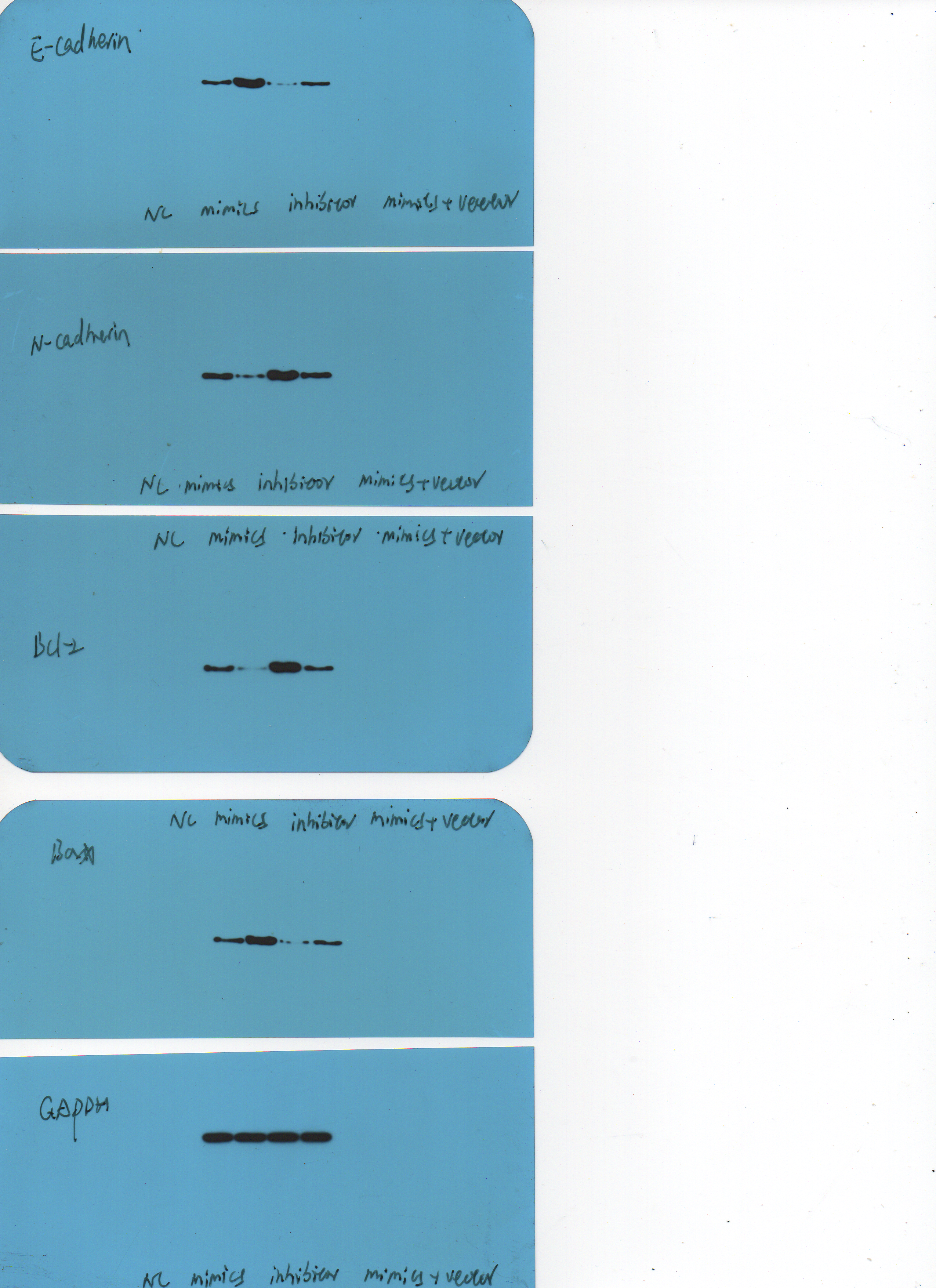

Supplement: Supplementary file 2 — Additional file 2. [file 12885_2020_6662_MOESM2_ESM.tif]

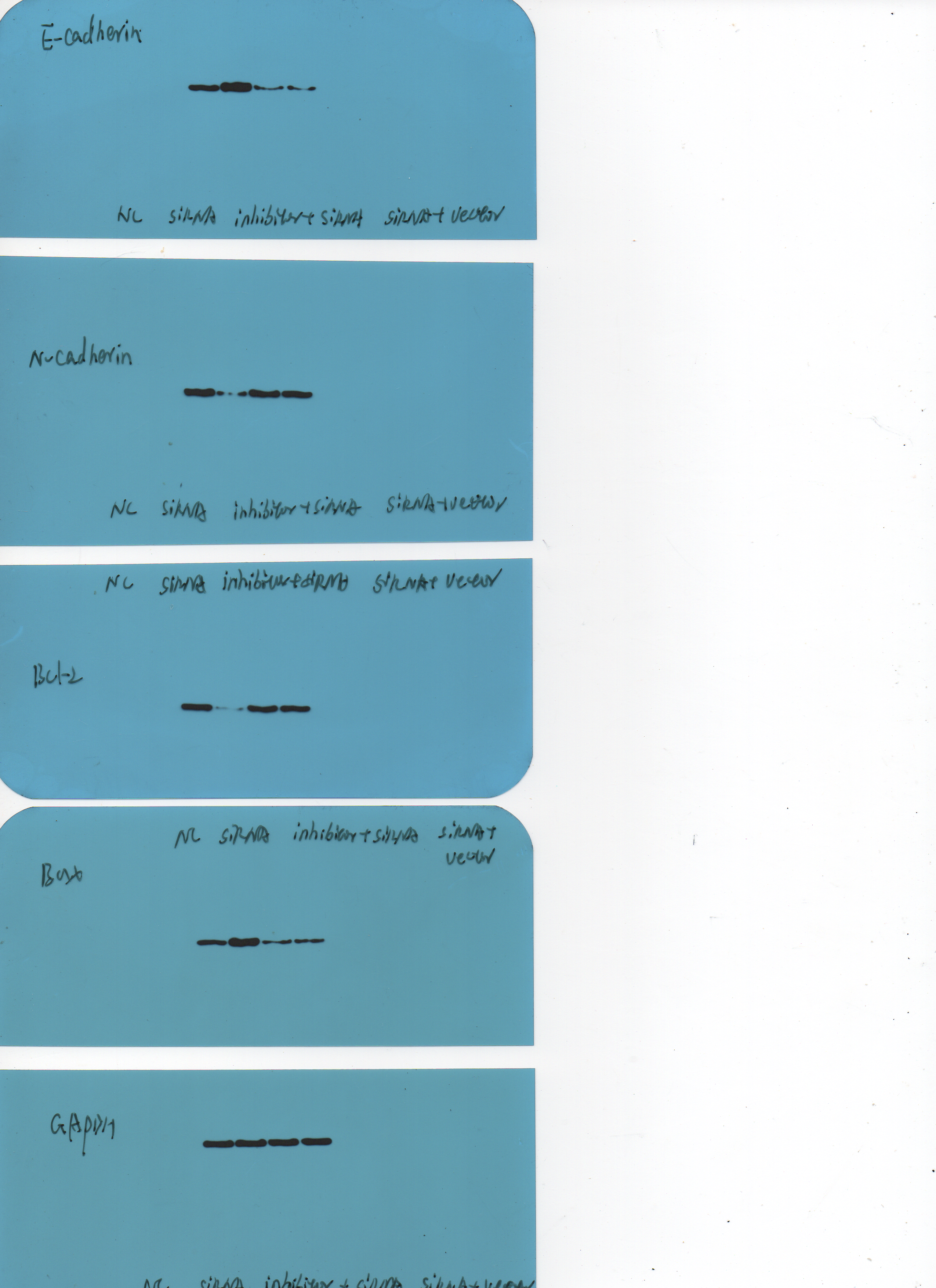

Supplement: Supplementary file 3 — Additional file 3. [file 12885_2020_6662_MOESM3_ESM.tif]
